# Conocimientos, actitudes y prácticas de teniasis y cisticercosis por *Taenia solium* en estudiantes y profesionales de bioanálisis en Maracay, Venezuela, 2020

**DOI:** 10.7705/biomedica.7668

**Published:** 2025-08-11

**Authors:** Glenda C. Rojas, María Aouad, Yumara Barrios, María M. Cortez

**Affiliations:** 1 Instituto de Investigaciones Biomédicas “Dr. Francisco J. Triana-Alonso”, Facultad de Ciencias de la Salud, Universidad de Carabobo, Aragua, Venezuela Universidad de Carabobo Facultad de Ciencias de la Salud Universidad de Carabobo Aragua Venezuela; 2 Departamento Clínico-Integral, Escuela de Bioanálisis, Facultad de Ciencias de la Salud, Universidad de Carabobo, Aragua, Venezuela Universidad de Carabobo Escuela de Bioanálisis Facultad de Ciencias de la Salud Universidad de Carabobo Aragua Venezuela; 3 Escuela de Bioanálisis, Facultad de Ciencias de la Salud, Universidad de Carabobo, Aragua, Venezuela Universidad de Carabobo Escuela de Bioanálisis Facultad de Ciencias de la Salud Universidad de Carabobo Aragua Venezuela

**Keywords:** Taenia solium, conocimientos, actitudes y práctica en salud, estudiantes, Venezuela., Taenia solium, health knowledge, attitudes, practice, students, Venezuela.

## Abstract

**Introducción.:**

La teniasis y la cisticercosis por *Taenia solium* ocasionan serios problemas económicos y de salud, y son prevalentes en Latinoamérica, África y Asia. Existen pocos estudios en que se evalúen los conocimientos, actitudes y prácticas (CAP) de los profesionales de la salud ante estas parasitosis.

**Objetivo.:**

Evaluar los conocimientos, las actitudes y las prácticas sobre *T. solium*, de los estudiantes y profesionales de bioanálisis, residentes en un área urbana no endémica para *T. solium* en Maracay (Aragua), región central de Venezuela.

**Materiales y métodos.:**

Se desarrolló un estudio transversal mediante la aplicación de una encuesta a estudiantes (n = 41) y profesionales (n = 41) del área de bioanálisis, para recopilar información sobre sus conocimientos, actitudes y prácticas con respecto a la teniasis y la cisticercosis.

**Resultados.:**

El 67,1 % de los participantes reconoció a *T. solium* como un problema de salud pública prevalente en las comunidades rurales; mientras que solo el 30,5 % acertó sobre el agente causal. Se evidenció un conocimiento limitado sobre la vía de transmisión de la teniasis (6,1 %), la cisticercosis humana (11 %) y la cisticercosis porcina (17,1 %). Asimismo, se observó un conocimiento reducido sobre los signos y síntomas de la teniasis (4,9 %) y las características de la carne de cerdo infectada con cisticercos de *T. solium* (3,7 %). Los profesionales mostraron un mayor conocimiento sobre teniasis, cisticercosis porcina, enfermedad grave por *T. solium* y neurocisticercosis (p < 0,05), mientras que los estudiantes se destacaron en la identificación del agente causal y en el método diagnóstico de elección para la teniasis (p < 0,05). La mayoría de los participantes mostró actitudes y prácticas positivas. Los profesionales fueron mejores en cuanto a la difusión de información sobre *T. solium* y en el manejo de pacientes infectados con *Taenia* spp. (p < 0,05).

**Conclusión.:**

Este estudio sobre los conocimientos, actitudes y prácticas de los profesionales y estudiantes de bioanálisis con respecto a *T. solium* en Venezuela, evidenció debilidades relacionadas con los aspectos clínico-epidemiológicos y diagnósticos. Se recomienda actualizar los conocimientos sobre *T. solium* y llevar a cabo más estudios relacionados, con muestras más amplias y en otras disciplinas.

## Abstract (en)

**Introduction.:**

Taeniasis and cysticercosis are diseases caused by *Taenia solium* prevalent in Latin America, Africa, and Asia, and associated with serious economic and health problems. To date, few studies have evaluated the knowledge, attitudes, and practices related to these parasitoses among health professionals.

**Objective.:**

To evaluate the knowledge, attitudes, and practices associated with *T solium* among students and professionals in the field of bioanalysis, residing in an urban, nonendemic area for *T solium* in Maracay, Aragua, central region of Venezuela.

**Materials and methods.:**

We conducted a cross-sectional study using a survey to collect data from students (n = 41) and professionals (n = 41) of bioanalysis regarding knowledge, attitudes, and practices related to taeniasis and cysticercosis in Maracay, Venezuela.

**Results.:**

Among all participants, 67.1% recognized *T. solium* as a public health problem prevalent in rural areas, while 30.5% correctly identified the causative agent. Limited knowledge was observed regarding the transmission of taeniasis (6.1%), human cysticercosis (11.0%), and porcine cysticercosis (17.1%), as well as the signs and symptoms of taeniasis (4.9%) and the characteristics of pork meat infected with *T. solium* cysticerci (3.7%). Professionals had greater knowledge of taeniasis, porcine cysticercosis, severe disease caused by T. solium, and neurocysticercosis (p < 0.05). Students knew better the causative agent and the preferred diagnostic method for taeniasis (p < 0.05). Most participants exhibited positive attitudes and practices. Professionals outperformed students in providing information regarding *T. solium* and dealing with patients infected with Taenia spp. (p < 0.05).

**Conclusion.:**

This study on knowledge, attitudes, and practices regarding *T. solium* among professionals and students of bioanalysis in Venezuela showed deficiencies in clinical- epidemiological and diagnostic aspects. We recommend updating knowledge on taeniasis and cysticercosis and developing further studies with larger sample sizes and the inclusion of other disciplines.

La teniasis y la cisticercosis son zoonosis causadas por el cestodo *Taenia solium*, endémicas y prevalentes en Latinoamérica, África y Asia. Sin embargo, los países desarrollados también pueden verse afectados debido a la inmigración, los viajes a regiones endémicas y el comercio internacional ^1^^,^^2^. La infección por *T. solium* se ha identificado como la principal causa de muerte por enfermedades transmitidas por alimentos, con una carga estimada de 2,8 millones de años de vida ajustados por discapacidad (AVAD) ^3^. El ciclo de vida de *T. solium* involucra principalmente al cerdo, que actúa como huésped intermediario del estadio larval; al ser humano, huésped definitivo de la forma adulta y, accidentalmente, también puede ser intermediario, y al medio ambiente, fuente de exposición a huevos infecciosos ^4^.

Cuando una persona sana consume carne de cerdo mal cocida e infectada con cisticercos, estas larvas se alojan en el intestino y se transforman en el estadio adulto (tenia). Posteriormente, se liberan proglótides cargadas de huevos o son eliminados en las heces del ser humano al medio ambiente. Estos huevos pueden ser ingeridos por los cerdos -quienes tienen hábitos coprofágicos-, lo que da lugar a la cisticercosis porcina; o por humanos, ya sea por contaminación o autoinfección ^5^, lo que ocasiona la cisticercosis humana ^1^^,^^6^.

La teniasis puede ser asintomática o causar manifestaciones clínicas menores. Su principal signo es la expulsión de proglótides y huevos. La sintomatología de la cisticercosis varía, dependiendo de la localización, el tamaño, el estado, el número de quistes y la respuesta inmunológica del huésped.

La localización de cisticercos en el sistema nervioso central, conocida como neurocisticercosis, es la forma clínica más grave de la enfermedad en humanos ^7^^,^^8^. La neurocisticercosis presenta una amplia heterogeneidad clínica, con manifestaciones clínicas que pueden incluir cefaleas, convulsiones, hidrocefalia, demencia u otros signos de lesiones intracraneales que ocupan espacio o puede ser asintomática y persistir durante muchos años ^9^^,^^10^. El cisticerco puede alojarse en el tejido cerebral (neurocisticercosis parenquimatosa) o en los espacios intraventriculares y subaracnoideos del cerebro y del cordón espinal (neurocisticercosis extraparenquimatosa). La primera es controlable y tiene buen pronóstico, mientras que la segunda es de peor pronóstico ^11^.

El control de estas parasitosis depende del diagnóstico temprano y el tratamiento oportuno de los individuos portadores. En el caso de *T. solium*, los individuos infectados con el cestodo adulto constituyen el foco de dispersión de huevos infecciosos; por lo tanto, la identificación y el tratamiento de los portadores es esencial para reducir la cisticercosis humana y la porcina ^11^.

El diagnóstico de teniasis se basa en el tamizaje coprológico (búsqueda y diferenciación de proglótides grávidas del cestodo adulto) y en el examen microscópico para la detección de huevos de *Taenia* spp. (también en muestras fecales del individuo). Otros métodos de diagnóstico incluyen la detección de anticuerpos anti-*Taenia*, el análisis de ADN o la identificación de coproantígenos en potenciales portadores ^11^^-^^14^.

El tratamiento se basa en la formulación de antihelmínticos (niclosamida y praziquantel) ^15^. Las diferentes formas de neurocisticercosis requieren diagnósticos y tratamientos especializados por parte de neurólogos y pueden incluir antihelmínticos, antiepilépticos, corticoesteroides e, incluso, cirugías ^16^.

Los criterios de diagnóstico incluyen estudios imagenológicos como la tomografía computarizada (TC) o la resonancia magnética (RM). La técnica de inmunoadsorción ligada a enzimas (ELISA) se usa para la detección serológica de los antígenos del cisticerco y sirve como apoyo diagnóstico en la cisticercosis. La identificación de los anticuerpos en suero es una prueba sugerente, pero carece de especificidad y no diferencia entre infecciones activas o previas. La prueba de inmunotransferencia enzimática con glucoproteínas de bajo peso molecular, afines a lectinas (EITB-LLGP), para anticuerpos séricos, sigue siendo el método de referencia ^12^ para identificar anticuerpos específicos de *T. solium* debido a su gran especificidad y sensibilidad. Sin embargo, su disponibilidad, al igual que la de la técnica ELISA, es limitada, lo que dificulta la detección y el tratamiento adecuado de los pacientes.

La cisticercosis porcina cursa con poca o ninguna sintomatología. Sin embargo, en algunos estudios se han reportado signos sugestivos de neurocisticercosis porcina ^17^. Para su diagnóstico, se pueden emplear métodos como la palpación lingual, la inspección de canales en el sitio del sacrificio o la revisión minuciosa del canal en otras necropsias. Se pueden usar pruebas serológicas comerciales, pero estas presentan datos insuficientes de sensibilidad y especificidad, además de reacciones cruzadas con la forma larvaria de *Taenia hydatigena* y, posiblemente, con otros parásitos ^12^^,^^18^.

La transmisión de *T. solium* en áreas rurales persiste principalmente por la defecación humana en el medio ambiente, la cría de cerdos libres y el consumo de carne de cerdo infectada con cisticercos o mal cocida. Además, no hay herramientas diagnósticas sensibles y de bajo costo que puedan usarse para hacer un diagnóstico a gran escala en zonas endémicas ^12^^,^^19^. No obstante, las intervenciones con participación de las comunidades pueden ser efectivas para reducir la incidencia y la prevalencia de la cisticercosis en algunos entornos de bajos recursos ^20^^,^^21^. Por su parte, las poblaciones urbanas difieren de las rurales respecto a patrones demográficos, hábitos de vida y carga de comorbilidades. Por lo tanto, los análisis basados en información recopilada de las poblaciones rurales no deberían extrapolarse a las poblaciones urbanas, sin la generación de evidencia que los respalde.

La prevalencia de *T. solium* en las comunidades urbanas se desconoce. La rápida expansión de comunidades urbanas y periurbanas -muchas de ellas en hacinamiento, sin acceso a agua potable, en viviendas insalubres, con cría de animales domésticos e inadecuada disposición de excretas humanas- propicia las condiciones favorables para la transmisión de *T. solium*, en conjunto con prácticas como la cría de cerdos en los traspatios, su matanza doméstica y el comercio informal de su carne. Además, la venta y el consumo de alimentos en las calles, a menudo en condiciones higiénicas deficientes, también inciden en la transmisión de *T. solium*^22^. Existe una clara necesidad de comprender mejor la presencia y el impacto del complejo teniasis-cisticercosis en el contexto urbano, incluyendo sus mecanismos de transmisión y los patrones demográficos asociados ^6^.

En zonas urbanas, uno de los problemas más importantes es el desplazamiento de individuos portadores de *T. solium* desde zonas endémicas o hacia ellas. Estas personas generalmente desconocen su condición de infectados y son un factor de riesgo en su entorno ^23^^-^^25^. Las estrategias propuestas para el control de *T. solium*, incluyen la administración masiva de drogas antihelmínticas a los humanos, el tratamiento y la vacunación profiláctica de cerdos y la educación sanitaria sobre teniasis y cisticercosis. No obstante, el éxito de las diferentes estrategias está influenciado por factores socioeconómicos, culturales, geográficos y ambientales ^26^. Para el éxito de estas medidas en teniasis, cisticercosis y otras parasitosis, es imprescindible conocer la información que posee la población en riesgo y los trabajadores de la salud, para diseñar e implementar intervenciones que promuevan cambios efectivos en el comportamiento (por ejemplo, reducción de riesgos y búsqueda de atención médica) ^27^.

La información sobre conocimientos, actitudes y prácticas relacionadas con *T. solium* es importante para establecer estrategias exitosas para el control del parásito. La identificación de vacíos en el conocimiento, patrones de comportamiento y creencias culturales puede facilitar la implementación de programas de control y estrategias de educación sanitaria que promuevan cambios de hábitos en las zonas rurales y urbanas ^28^^,^^29^.

La información sobre conocimientos, actitudes y prácticas relacionadas con *T. solium* de los profesionales del área de la salud es escasa. En Estados Unidos, se evaluó el conocimiento sobre *T. solium* de más de 1.000 ginecoobstetras ^30^. Aunque muchos contaban con algunas nociones, había confusiones respecto al ciclo de vida del parásito, los mecanismos de transmisión de la infección y la enfermedad en general. En Tanzania, se encontró insuficiente capacidad de diagnóstico y poco conocimiento sobre *T. solium* por parte de trabajadores de la salud, como funcionarios de atención primaria e inspectores veterinarios ^12^.

En Venezuela, aunque el binomio teniasis-cisticercosis está documentado ^31^^-^^33^, hay poca información sobre los conocimientos, actitudes y prácticas relacionadas con *T. solium* que puedan tener los profesionales de la salud y la población en general. En un estudio sobre neurocisticercosis en el que se evaluaron 221 habitantes del estado Mérida (Venezuela) se evidenció un gran desconocimiento de la enfermedad: el 100 % de los residentes en las áreas rurales la desconocía, así como el 89 % de los residentes de zonas urbanas y el 80 % de los estudiantes de octavo semestre de medicina ^34^. En el estado Aragua se han diagnosticado casos de neurocisticercosis en adultos provenientes de Maracay y zonas cercanas ^35^^-^^37^, lo cual sugiere la potencial circulación de *T. solium*, la presencia de factores de riesgo y su transmisión local. También, se han referido casos de *T. saginata* en algunas zonas urbanas y periurbanas de Aragua ^38^, lo que podría reflejar deficiencias en la inspección sanitaria de los animales para el consumo humano.

Los profesionales de bioanálisis en Venezuela son egresados universitarios del área de la salud, dedicados al análisis de muestras biológicas (humanas y veterinarias) y que contribuyen a la solución de situaciones sanitarias en su área de influencia, mediante la generación de datos diagnósticos, la prevención y el tratamiento de enfermedades, y la promoción de la salud ^39^. La evaluación de los conocimientos, actitudes y prácticas relacionadas con *T. solium* en esta población, indicaría su capacidad para contribuir al control de las parasitosis causadas por este agente mediante su diagnóstico, el asesoramiento a los potenciales portadores y la inclusión de las comunidades en la generación de estrategias de control.

Por su rol en el análisis epidemiológico, parasitológico y serológico, el objetivo de este trabajo fue evaluar los conocimientos, actitudes y prácticas sobre *T. solium* de los profesionales y estudiantes de bioanálisis residentes del área urbana no endémica de Maracay (Aragua), en la región central de Venezuela.

## Materiales y métodos

Se llevó a cabo un estudio descriptivo de corte transversal basado en la información recopilada mediante una encuesta sobre conocimientos, actitudes y prácticas relacionadas con *T. solium*. Se utilizó un cuestionario diseñado según la literatura reportada ^30^^,^^40^, el cual fue evaluado por tres especialistas: uno de salud pública, uno de parasitología y uno de epidemiología. Antes de su aplicación, se hizo una prueba piloto con diez individuos universitarios para evaluar la comprensión, la claridad y la facilidad de las preguntas propuestas.

El cuestionario constaba de cuatro secciones:

- datos demográficos: nombre (opcional), cargo, edad, sexo y nivel académico;
- evaluación de conocimientos: 20 preguntas sobre vías de transmisión, manifestaciones clínicas y control de *T. solium*. Siete de las preguntas eran cerradas y tenían tres opciones de respuesta (sí / no / no responde), y 13 preguntas eran abiertas y exigían la respuesta en un breve texto;
- evaluación de actitudes: preguntas sobre la disposición del encuestado para adoptar conductas de prevención y control de *T. solium* (dos preguntas cerradas y cuatro abiertas), y
- evaluación de prácticas: siete preguntas cerradas sobre hábitos personales relacionados con riesgos de infección por *T. solium*.

La aplicación del cuestionario fue dirigida por investigadores y estudiantes, y se realizó mediante entrevistas presenciales de una hora, aproximadamente, en los sitios usuales de actividad de los participantes (laboratorios y aulas de clase).

### Población participante

La población del estudio estuvo integrada por 82 individuos: 41 estudiantes de 5^o^ año del programa de Bioanálisis de la Facultad de Ciencias de la Salud de la Universidad de Carabobo, sede Aragua, y 41 profesionales egresados del mismo programa que ejercen en los laboratorios del municipio Girardot del estado de Aragua. No se calculó la muestra, ya que la idea fue conseguir el mayor número posible de estudiantes y profesionales en el sitio de estudio.

### Criterios de inclusión y exclusión

La selección de los sujetos se hizo al azar, según la disposición para participar en el estudio de forma voluntaria y anónima. Los criterios de inclusión fueron: participación voluntaria, ser estudiante activo del último año de la carrera o ser profesional activo de bioanálisis. El criterio de exclusión fue la manifestación voluntaria de no participar en el estudio.

### Localidad

El estudio fue realizado entre enero del 2020 y enero del 2021 en la zona urbana de Maracay (10°14’49” N; 67°35’45” O), en el estado de Aragua, ubicado en la región costera centro-norte de Venezuela. El trabajo de campo fue realizado por estudiantes y profesores del Departamento Clínico Integral, Escuela de Bioanálisis, Facultad de Ciencias de la Salud, Universidad de Carabobo, sede Aragua (FCS-UCSA).

### Evaluación de respuestas

Las respuestas a las preguntas abiertas sobre conocimientos relacionados con *T. solium*, se clasificaron como “correctas”, “incorrectas”, “vagas” o “no responde”. Las respuestas sobre actitudes se evaluaron como “actitud positiva”, según su tendencia hacia la prevención del riesgo de infección por *T. solium*. Finalmente, todas las respuestas sobre prácticas se aceptaron, ya que representaban los hábitos de los participantes y su potencial impacto en la transmisión de *T. solium*.

### Análisis estadístico

Las encuestas respondidas por los participantes fueron revisadas para contabilizar el número de respuestas correctas por grupo evaluado. La información recopilada sobre conocimientos, actitudes y prácticas, se ingresó en una base de datos creada en Microsoft Excel®. Los datos se analizaron con el *software* SPSS®, versión 13.0. Se calcularon las frecuencias absolutas y relativas (porcentajes) de las respuestas para cada una de las preguntas. El análisis estadístico comparativo entre grupos (estudiantes y profesionales) se llevó a cabo mediante la prueba de ji al cuadrado. Las diferencias en las respuestas de los dos grupos se consideraron estadísticamente significativas, si el valor de p era menor de 0,05.

### Consideraciones éticas

El proyecto fue aprobado por el Departamento Clínico Integral de la Escuela de Bioanálisis de la FCS-UCSA. La presente investigación no implicó riesgos de salud para los participantes, ya que únicamente se emplearon métodos documentales y no hubo intervención alguna sobre los sujetos del estudio. A los participantes se les garantizó la privacidad, la confidencialidad y el anonimato de la información.

Antes de la aplicación de la encuesta, a los participantes se les explicó el protocolo de la investigación y los posibles beneficios que podrían derivarse de la misma. Todos los participantes manifestaron verbalmente su voluntad de participar y escribieron personalmente, sin ningún tipo de ayuda externa, las respuestas que consideraron pertinentes. Los participantes no recibieron ninguna compensación económica. Se dejó constancia de que la decisión de no participar en el estudio no acarrearía ningún tipo de inconvenientes y que, en el futuro, podrían participar en cualquier tipo de evento derivado del presente proyecto.

## Resultados

Se incluyeron 82 adultos distribuidos en dos grupos. En el grupo 1, se incluyeron 41 estudiantes: 34/41 (82,9 % mujeres) y 6/41 (14,6 % hombres) que cursaban el quinto año de bioanálisis; 1 de 41 (2,5 %) no respondió; 36 de 41 (87,8 %) estaba en el rango de edad de 21 a 30 años y, 1 de 41 (2,4 %); en el de 30 a 40 años, 4 de 41 (9,8 %) no respondieron. En el grupo 2, se incluyeron 41 profesionales: 25 de 41 (61,0 %) eran mujeres y 12 de 41 (9,8 %) hombres; el 29,2 % no respondió; según el rango de edad, 13 de 41 (31,7%) estaba entre los 21 y los 30 años; 8 de 41 (19,5 %), entre los 31 y los 40 años, y 8 de 41 (19,5 %) entre los 41 y los 50 años; 12 de 41 (29,3 %) no respondieron. Todos los participantes desarrollaban sus actividades en Maracay, municipio Girardot, del estado Aragua, y residían en Maracay o Turmero, municipio Mariño, ambas zonas urbanas colindantes del estado Aragua.

### Conocimientos

Las respuestas sobre el conocimiento de la infección por *T. solium*, se muestran en los cuadros 1 y 2. En el cuadro 1 (preguntas cerradas), se deduce que la mayoría de los encuestados tenía buenos conocimientos: el 89 % dijo conocer sobre la teniasis y la cisticercosis, el 95,1 % afirmó que esta parasitosis puede causar una enfermedad grave y que sabía qué es la neurocisticercosis, y el 76,8 % sabía que la cisticercosis puede afectar a los cerdos. Del grupo de estudiantes, el 87,8 % sabía que la cisticercosis puede afectar a los cerdos y menos del 10 % afirmó que esta se transmite de persona a persona (p < 0,05). Por su parte, los profesionales manejaron mejor el concepto de neurocisticercosis (p < 0,05).

**Cuadro 1.** Conocimientos sobre teniasis y cisticercosis por *Taenia solium* en estudiantes y profesionales de bioanálisis entre el 2020 y el 2021 en Maracay, Venezuela

Pregunta	Total (n = 82)	Estudiantes (n = 41)	Profesionales (n = 41)	P(χ^2^ *)*
		n (%)		
¿Conoce la teniasis o la cisticercosis?	73 (89,0)	37 (90,2)	36 (87,8)	0,4691^ns^
¿La teniasis o la cisticercosis pueden causar enfermedad grave?	78 (95,1)	37 (90,2)	41 (100,0)	0,1221^ns^
¿Sabe qué es la neurocisticercosis?	75 (91,5)	34 (82,9)	41 (100,0)	0,0218*
¿La cisticercosis puede afectar a los cerdos?	63 (76,8)	36 (87,8)	27 (65,9)	0,0479*
¿Conoce la forma de infección de los cerdos?	42 (51,2)	17(41,5)	25 (61,0)	0,2071^ns^
¿Reconoce carne de cerdo infectada con cisticercos?	22 (26,8)	11 (26,8)	11 (26,8)	0,0141*
¿La cisticercosis se transmite de persona a persona?	16(19,5)	4 (9,8)	12(29,3)	0,0423*

* diferencia significativa

ns: diferencia no significativa; estudiantes: alumnos de quinto año de bioanálisis; profesionales: licenciados en bioanálisis

**Cuadro 2.** Conocimientos sobre teniasis y cisticercosis por *Taenia solium* en estudiantes y profesionales en bioanálisis entre el 2020 y el 2021 en Maracay, Venezuela

Pregunta	Total (n = 82)	Estudiantes Profesionales (n = 41) (n = 41)		**P(*X*** ^2^ **)**
		n (%)		
¿Cuál es el agente causal de la teniasis y la cisticercosis?	25 (30,5)	14(34,1)	11 (26,8)	0,0363*
¿Cómo se adquiere la teniasis?	5(6,1)	2 (4,9)	3 (7,3)	0,0104*
¿Cómo se adquiere la cisticercosis humana?	9(11,0)	7(17,1)	2 (4,9)	0,2325^ns^
¿Cuál es la forma de infección de los cerdos?	14(17,1)	5(12,2)	9 (22,0)	0,0441*
¿Cuáles son las características de la carne de cerdo con cisticercos?	3 (3,7)	2 (4,9)	1 (2,4)	0,2567^ns^
¿La teniasis o la cisticercosis son un problema de salud en personas de zona rural o urbana?	55 (67,1)	25 (61,0)	30 (73,2)	0,1616^ns^
Especifique cuál enfermedad grave es causada por teniasis o cisticercosis	27 (32,9)	11 (26,8)	16(39,0)	0,0369*
Defina la neurocisticercosis.	22 (26,8)	2 (4,9)	20 (48,8)	0,0001**
¿Cuáles son los signos y síntomas de la cisticercosis humana?	35 (42,7)	14(34,1)	21 (51,2)	0,2229^ns^
¿Cuáles son los medicamentos genéricos para tratar la cisticercosis?	19(23,2)	4 (9,8)	15(36,6)	0,0835^ns^
¿Cuáles son los signos y síntomas de la teniasis?	4 (4,9)	1 (2,4)	3 (7,3)	0,1189^ns^
¿Cuáles son los medicamentos genéricos para tratar la teniasis?	6 (7,3)	2 (4,9)	4 (9,8)	0,3707^ns^
¿Cuál es el método de elección para el diagnóstico de la teniasis?	47 (57,3)	28 (68,3)	19(46,3)	0,0001**

^n^ ^s^ Diferencia no significativa; estudiantes: alumnos de quinto año de bioanálisis; profesionales: licenciados en bioanálisis

* diferencia significativa

** diferencia muy significativa

En relación con el reconocimiento de la carne de cerdo infectada con cisticercos, la proporción de individuos que respondió afirmativamente fue igual en ambos grupos (26,8 %). Sin embargo, 27 de 41 (65,9 %) estudiantes manifestaron no reconocer la carne de cerdo infectada, en comparación con 15 de 41 (36,6 %) de los profesionales, diferencia que resultó ser significativa (p <0,05) (valores no incluidos en el cuadro); además, 15 de 41 (36,6 %) profesionales y 3 de 41 (7,3 %) estudiantes no respondieron dicha pregunta (valores no incluidos en el cuadro). En el cuadro 2 se muestran las respuestas a las preguntas abiertas sobre conocimientos de *T. solium*.

La mayoría (67,1 %) de los participantes respondieron correctamente que la teniasis y la cisticercosis son un problema de salud pública que afecta a las zonas rurales y urbanas, aunque predomina en las primeras. El 57,3 % conocía los métodos de elección para el diagnóstico de la teniasis, pero sólo el 42,7 % sabía los signos y síntomas de la cisticercosis humana; el 32,9 % respondió cuál es la enfermedad grave que ocasiona la teniasis y la cisticercosis, y el 30,5 % identificó correctamente el agente causal.

Finalmente, solo el 17,1 % acertó las respuestas sobre las vías de transmisión de la cisticercosis en cerdos y apenas el 11 % respondió correctamente sobre la transmisión de la cisticercosis en humanos; el 6,1 % de los participantes conocía las vías de transmisión de la teniasis y, el 4,9 %, sus signos y síntomas; el 3,7 % de la población incluida describió acertadamente las características de la carne de cerdo infectada con cisticercos de *T. solium*. Un mayor número de estudiantes (14/41; 34,1%) sabía el agente causal (p < 0,05) y el método de elección para el diagnóstico de la teniasis (p < 0,05). Por su parte, el grupo de profesionales respondió correctamente cómo se adquieren la teniasis (p <0,05) y la cisticercosis porcina (p < 0,05), cuál es la enfermedad grave ocasionada por *T. solium* (p <0,05) y qué es la neurocisticercosis (p < 0,05).

### Actitudes

Las preguntas del cuadro 3 estaban orientadas a evaluar la actitud positiva (prevención) o negativa (riesgo) del individuo en situaciones que pudieran favorecer la infección por *T. solium*. Ambos grupos de participantes respondieron afirmativamente (preguntas cerradas) a la necesidad de brindar más información al público sobre *T. solium* (91,5 %) y manifestaron compartir información sobre la transmisión de esta parasitosis con sus allegados (70,7 %).

**Cuadro 3.** Actitudes sobre teniasis y cisticercosis por *Taenia solium* en estudiantes y profesionales de bioanálisis entre el 2020 y el 2021 en Maracay, Venezuela

Pregunta	Total (n = 82)	Estudiantes (n = 41)	Profesionales (n = 41)	p(X^2^)
		n (%)		
¿Considera que el público general necesita información sobre teniasis o cisticercosis?	75 (91,4)	37 (90,2)	38 (92,7)	0,9249^ns^
¿Ha informado a sus allegados sobre cómo se transmite la teniasis o la cisticercosis?	58 (70,7)	22 (53,7)	36 (87,8)	0,0074*
¿Cuál es su proceder ante un familiar que está comiendo carne de cerdo mal cocida?	78 (95,1)	37 (90,2)	41 (100,0)	0,1222^ns^
¿Cuál es su proceder como bioanalista ante un paciente infectado por *Taenia* spp.?	61 (74,4)	23 (56,1)	38 (92,7)	0,0020*
¿Cómo se puede prevenir la transmisión de teniasis o cisticercosis en comunidades rurales?	65 (79,3)	27 (65,9)	38 (92,7)	0,0244*
¿Cómo se puede prevenir la transmisión de teniasis o cisticercosis en comunidades urbanas?	68 (82,9)	31 (75,6)	37 (90,2)	0,208^ns^

ns: diferencia no significativa; estudiantes: alumnos de quinto año de bioanálisis; profesionales: licenciados en bioanálisis

* diferencia significativa

En las preguntas abiertas, todos los participantes mostraron una actitud positiva en situaciones de riesgo, por ejemplo, en el caso de que un familiar estuviera consumiendo carne de cerdo mal cocida (95,1 %); o ante un paciente con una muestra fecal con presencia de *Taenia* spp. (74,4 %). Los participantes también respondieron positivamente respecto a la prevención de la transmisión de *T. solium* en comunidades rurales (79,3 %) y urbanas (82,9 %).

Los profesionales mostraron una mayor actitud positiva para informar sobre *T. solium* a sus allegados (p < 0,05), sobre cómo proceder ante un paciente infectado con huevos de *Taenia* sp. (p < 0,05) y cómo prevenir estas parasitosis en zonas rurales (p < 0,05).

### Prácticas

En el cuadro 4, se muestran los resultados de la evaluación de prácticas de riesgo o medidas preventivas relacionadas con la transmisión de *T. solium*. En las respuestas a estas preguntas se consideraron los hábitos de los participantes que podrían influir o no influir en una infección por *T. solium*. Setenta y seis de 82 (92,7 %) de los encuestados manifestó consumir carne de cerdo, cocida (n = 71) o bien cocida (n = 4) 75 de 82 individuos (98,7%), y solo el 14,6 % reconoció comprar carne de cerdo en ventas ambulantes. Respecto al lavado de manos, la mayoría contestó que lo hacía después de ir al baño (98,8 %), antes de comer (97,6 %) y antes de preparar alimentos (98,9 %). El 98,8 % acostumbraba lavar las verduras y las frutas antes de comerlas. Respecto al consumo de agua, el 48,8 % de los entrevistados refirió beber agua potable (comercialmente embotellada) y, en menor proporción, clorada (3,7 %) (tratamiento de cloración adicional, ya que teóricamente el agua de las tuberías ya está clorada), filtrada (14,6 %), hervida (11,0 %) y del grifo (2,4 %). El 17,1 % respondió que consumía varios tipos o “combinaciones” de agua. Finalmente, solo la mitad (48,8 %) de los participantes informó reportar casos positivos -con presencia de huevos de *Taenia* spp - a organismos oficiales. No hubo diferencias significativas entre los grupos estudiados.

**Cuadro 4.** Prácticas sobre teniasis y cisticercosis por *Taenia solium* en estudiantes y profesionales de bioanálisis entre el 2020 y el 2021 en Maracay, Venezuela.

Pregunta		Total	Estudiantes	Profesionales
		(n = 82)	(n = 41)	(n = 41)
			n (%)	
¿Cómo consume la carne de cerdo?		76 (92,7)	39 (95,1)	37 (90,2)
	Cruda	1 (1,3)	1 (2,6)	0 (0,0)
	Semicruda	0 (0,0)	0 (0,0)	0 (0,0)
	Cocida	4 (5,3)	4(10,3)	0 (0,0)
	Bien cocida	71 (93,4)	34 (87,2)	37 (100,0)
¿Compra carne de cerdo cruda o cocida en ventas ambulantes?		12(14,6)	8(19,5)	4 (9,8)
Usted se lava las manos:				
	Después de ir al baño	81 (98,8)	40 (97,6)	41 (100)
	Antes de comer	80 (97,6)	39 (95,1)	41 (100)
	Antes de preparar alimentos	81 (98,8)	40 (97,6)	41 (100)
¿Lava correctamente las verduras y las frutas antes de comerlas?		81 (98,8)	40 (97,6)	41 (100)
Agua de consumo:				
	Potable	40 (48,8)	22 (53,7)	18(43,9)
	Clorada	3 (3,7)	0 (0,0)	3 (7,3)
	Filtrada	12(14,6)	8(19,5)	4 (9,8)
	Hervida	9(11,0)	2 (4,9)	7(17,1)
	De grifo	2 (2,4)	1 (2,4)	1 (2,4)
	Combinaciones	14(17,1)	8(19,5)	6(14,6)
	No responde	2 (2,4)	0 (0,0)	2 (4,9)
¿Reporta casos positivos de *Taenia* sp. al sistema de salud pública?		40 (48,8)	20 (48,8)	20 (48,8)

Estudiantes: alumnos de quinto año de bioanálisis; profesionales: licenciados en bioanálisis

## Discusión

La prevención y el manejo de la teniasis humana son esenciales para el control de la cisticercosis y la reducción de la carga mundial de epilepsia ^41^. Este control puede lograrse por: (i) la introducción inmediata de condiciones sanitarias adecuadas y (ii) la implementación de actividades de vigilancia sanitaria. Si bien la primera no es viable a corto plazo, la segunda opción -vigilancia sanitaria- sería el enfoque más viable para el control de *T. solium*. Dicha vigilancia consiste en identificar portadores del parásito en estadio adulto y recae sobre los profesionales de la salud. En Venezuela, los profesionales de bioanálisis son los encargados de analizar muestras biológicas humanas, de ahí la importancia de identificar sus conocimientos, actitudes y prácticas relacionados con *T. solium*. Hasta la fecha, este es el primer estudio en el que se evalúan tales aspectos en estos profesionales.

El estudio se llevó a cabo en 82 estudiantes y profesionales de bioanálisis. En ambos grupos predominaron las mujeres; la mayoría de los estudiantes estuvo en el grupo etario de 21 a 30 años (87 %), mientras que los profesionales abarcaban un rango de edad más amplio (31,7 % á 30 años, 39 % entre 31 y 50 años). Resultados similares según sexo y edad, se reportaron en estudios de conocimientos, actitudes y prácticas sobre leishmaniasis y toxoplasmosis entre estudiantes de medicina, veterinaria y biología ^42^^,^^43^; y sobre esquistosomiasis y leishmaniasis, en trabajadores de la salud (enfermeras, médicos) ^27^^,^^44^. Aunque consultar la literatura científica o preguntar a otros colegas es una práctica común en las profesiones médicas ^45^, los participantes respondieron la encuesta sin usar ningún tipo de información o ayuda externa. El estudio se realizó durante el período de la pandemia de COVID-19, lo que dificultó contactar y entrevistar a las personas, dadas las restricciones de transporte y la suspensión de actividades en la universidad y la ciudad en general. Esta circunstancia incidió en el bajo número de participantes.

Los resultados sobre conocimientos de *T. solium*, evaluados por preguntas cerradas y abiertas, mostraron discrepancias entre sí: 5 de 7 preguntas cerradas fueron respondidas correctamente por más del 50 % de los participantes, lo que sugiere un buen conocimiento (cuadro 1), mientras que solo 2 de las 13 preguntas abiertas (cuadro 2) fueron respondidas acertadamente por cerca de la mitad de los participantes, lo que indica poco conocimiento sobre el parásito. Estas diferencias podrían deberse a que las preguntas cerradas ofrecen directamente las opciones de respuestas (sí / no/ no responde), mientras que las preguntas abiertas no lo hacen. Aunque estas últimas son más difíciles de codificar y analizar, proporcionan más información textual -como opiniones, justificaciones o información espontánea- lo que podría evidenciar vacíos de conocimiento o confusión en el encuestado ^46^.

Solo la minoría de los participantes mostró conocimientos deficientes sobre cómo se adquiere la cisticercosis humana (9/82; 11 %) y la porcina (14/82; 17,1 %), los signos y síntomas de la teniasis (4/82; 4,9 %), y las características de la carne de cerdo infectada con cisticercos de *T. solium* (3/82; 3,7 %). Aproximadamente, una tercera parte (25/82; 30,5%), de los participantes identificó el agente causal y 22 de 82 (26,8 %) conocía la definición de neurocisticercosis. Los estudiantes mostraron mayor conocimiento sobre el agente causal y el método de elección para el diagnóstico de la teniasis (p < 0,05), posiblemente por sus recientes cursos curriculares de parasitología.

Anteriormente, en Venezuela, se había reportado un importante desconocimiento (80 %) de la neurocisticercosis en estudiantes de octavo semestre de medicina ^34^. En estudios de Marruecos con estudiantes de medicina, veterinaria y biología, se reportó que menos de la mitad de los participantes había oído sobre toxoplasmosis (42,6 %), su agente causal (36,5 %) o su huésped definitivo (32,1 %) ^42^^,^^47^, o sobre equinococcosis quística ^48^. Aunque son parasitosis diferentes y estudiantes de otras disciplinas, estos resultados fueron similares a los obtenidos en el presente estudio. Otros estudios semejantes en médicos han referido conocimientos pobres a moderados sobre dengue, enfermedad de Chagas y rickettsiosis ^49^.

En Estados Unidos, se encontró que los gineco-obstetras tenían conocimientos limitados sobre la transmisión de la teniasis (31,4 %) y la cisticercosis (14,5 %), y sobre los riesgos de cisticercosis en recién nacidos de madres con teniasis (30,3 %) ^30^. En trabajadores sanitarios (médicos, enfermeras y parteras), también se observaron conocimientos deficientes sobre los vectores de la leishmaniasis ^44^.

Respecto a las actitudes frente a situaciones de riesgo que involucran posible transmisión de *T. solium*, este estudio demuestra que, aun con las lagunas de conocimientos antes señaladas, la mayoría de los encuestados tenía una actitud positiva (cuadro 3) hacia la vigilancia y la prevención de infecciones por el parásito. Estas acciones incluían brindar información relacionada con teniasis y cisticercosis al público, orientación a pacientes infectados con huevos de *Taenia* spp. y difusión de medidas de prevención en áreas rurales o urbanas.

Los participantes reconocieron la necesidad de actualizar la información disponible sobre teniasis y cisticercosis. Los profesionales mostraron una mejor actitud hacia situaciones de riesgo para estas parasitosis posiblemente por su experiencia en el trato de pacientes afectados por diferentes enfermedades, a quienes se les informa dónde pueden conseguir el tratamiento adecuado. Este resultado concuerda con el mayor conocimiento y mejor percepción sobre la leishmaniasis reportado entre profesionales de la salud ^43^. No obstante, en otros estudios se han reportado actitudes negativas -como desinterés, falta de observancia de las guías establecidas o actitudes estigmatizantes ^50^^,^^51^. Entre estudiantes universitarios de diferentes disciplinas, los formados en parasitología demostraron mejores actitudes y prácticas frente a enfermedades parasitarias transmitidas por alimentos ^52^.

En general, se observaron buenos hábitos higiénicos frente a situaciones de riesgo para teniasis y cisticercosis. Los resultados demostraron un buen grado de observancia de las prácticas de prevención de infecciones por *T. solium* y otros patógenos. Muy pocas personas (14,6 %) compraban carne de cerdo en ventas ambulantes y casi todos los participantes consumían la carne de cerdo cocida y bien cocida, e indicaron lavar las frutas y vegetales antes de su consumo.

En el presente estudio, las características de la población estudiada -individuos con educación universitaria, residentes de zonas urbanas- posiblemente incidieron en sus buenas prácticas sanitarias. Asimismo, la coyuntura de la pandemia de COVID-19 puede haber influido positivamente en los resultados de las prácticas, ya que, durante ese periodo, la población en general -y especialmente los profesionales sanitarios- fueron sometidos a intensas campañas públicas y privadas sobre higiene. De forma similar, en un estudio en China, los médicos mostraron mayor atención a la seguridad alimentaria después de la pandemia de COVID-19 ^53^.

Los resultados de los estudiantes concuerdan con un estudio sobre equinococosis en el que la población estudiada tenía la actitud correcta, a pesar de los conocimientos limitados ^47^. De hecho, sus respuestas relacionadas con las prácticas fueron mejores que las reportadas por estudiantes de enfermedades parasitarias transmitidas por alimentos ^54^. Algo parecido se documentó en un estudio con médicos y enfermeras en Etiopía, en el que se observaron prácticas deficientes en el lavado de manos para la prevención de la tuberculosis; y en un reporte de teniasis causada por *T. saginata*, en trabajadores de la industria cárnica, asociada con el consumo de carne cruda, la matanza de animales en patios traseros y la defecación en el medio ambiente. Asimismo, se evidenció un menor cumplimiento de las prácticas para la prevención de infecciones nosocomiales, así como poca observancia de la guía nacional para el diagnóstico y el tratamiento de la malaria, y la prevención de otras infecciones en trabajadores sanitarios ^49^^,^^55^^-^^57^.

Es importante mencionar el escaso reporte (48,8 %) de casos positivos (presencia de huevos de *Taenia* spp.) a los entes de salud pública. Algunos participantes argumentaron que no existen protocolos para tales reportes. En Venezuela, al igual que en otros países, el reporte de estos casos se deja a voluntad y criterio del profesional a cargo del laboratorio. Cabe mencionar que el reporte de estos casos es clave para que el departamento de salud pública identifique posibles portadores de *T. solium*, supervise sus actividades (por ejemplo, manipulación de alimentos destinados al consumo público) y, de este modo, contribuya a disminuir el riesgo de transmisión de cisticercosis en el grupo familiar y la comunidad. Una mejor colaboración entre laboratorios, clínicas privadas y departamentos de salud pública puede prevenir la neurocisticercosis mediante la identificación efectiva y temprana de portadores de *T. solium* y la administración oportuna del tratamiento adecuado ^58^.

Entre las debilidades para el desarrollo de programas de control de *T. solium*, están la escasez de datos epidemiológicos válidos, que no existen vacunas para *T. solium* y que hay un desconocimiento sobre *T. solium* en la población afectada y en riesgo ^59^. Por lo anterior, es indudable que el desempeño calificado de los bioanalistas debe ser una de las primeras líneas de vigilancia para promover el cuidado de la salud humana, además de ser una herramienta primordial para el desarrollo y la implementación de estrategias de control para estas parasitosis ^7^^,^^19^^,^^41^. Esto enfatiza la relevancia del entrenamiento y la actualización de los profesionales sanitarios en las parasitosis endémicas de Venezuela, para lograr un diagnóstico temprano y registrar los casos sospechosos o confirmados de infecciones por *T. solium*^53^.

Finalmente, para enfrentar las enfermedades zoonóticas y otras situaciones de interés global, debe existir una estrecha colaboración entre los profesionales de la salud y afines, que involucre a estudiantes y profesionales de diversas disciplinas. Se requiere el liderazgo de profesores y altos directivos en los diferentes campos de la salud para compartir conocimientos y articular actividades enfocadas en programas de control de parásitos y otros patógenos ^60^^,^^61^.

Este estudio presenta limitaciones como un pequeño tamaño de muestra, el tipo de cuestionario empleado y la restricción de la zona estudiada. Sin embargo, se logró identificar debilidades en el conocimiento sobre *T. solium* en la población evaluada, resaltar la necesidad de realizar actividades de actualización y entrenamiento contra dicho parásito para fortalecer el conocimiento básico de las comunidades en general y prevenir esta parasitosis; además, constituye una línea de base para estudios similares. Se recomienda extender el estudio hacia zonas rurales y endémicas y también a otros trabajadores de la salud (médicos y enfermeras) y otros actores, como maestros y líderes comunitarios.

En conclusión, según lo reportado en la literatura revisada, este es el primer estudio sobre conocimientos, actitudes y prácticas relacionadas con *T. solium* en estudiantes y profesionales de bloanálisis en Venezuela. En este trabajo, se observaron brechas en el conocimiento sobre *T. solium* en la población general encuestada, particularmente, en aspectos clínico-epidemiológicos y diagnósticos. Se encontraron diferencias puntuales entre el grado de conocimiento de profesionales y estudiantes. El desconocimiento de estos aspectos de *T. solium* puede resultar en fallas en el diagnóstico y, por lo tanto, en que los portadores del parásito adulto no reciban el tratamiento oportuno y adecuado. En consecuencia, podría aumentar el riesgo de infección por *T. solium* en la familia, la comunidad y el personal de salud. Por esta razón, se necesita una actualización de la información disponible para fortalecer el conocimiento sobre *T. solium* en profesionales de la salud.

## Archivos suplementarios

Anexo 1. Evaluación de conocimientos, actitudes y prácticas sobre teniasis y cisticercosis en estudiantes y profesionales de bioanálisis de la Universidad de Carabobo de la sede Aragua

Instrucciones: Marque una “X” en las preguntas con opciones de Sí y No, o responda de forma breve y concisa. Si no conoce la respuesta, ignore la pregunta y continúe con la siguiente. Agradecemos su participación.

Encuesta No.:_ Fecha:__Lugar:__

Edad:__Sexo:__Año de egreso:

Dirección de habitación: _______________________

Nombre (opcional)____________________________

Parte I: conocimientos sobre el binomio teniasis-cisticercosis

¿Conoce usted la teniasis y la cisticercosis? Sí_No_

¿La teniasis o cisticercosis pueden causar una enfermedad grave?

Sí_No_

¿Sabe qué es la neurocisticercosis? Sí_No_

¿La cisticercosis puede afectar a los cerdos? Sí_No_

¿Conoce la forma de infección del cerdo? Sí_No_

¿Reconoce la carne de cerdo infectada con cisticercos? Sí_No_

¿La cisticercosis puede transmitirse de persona a persona? Sí_No_

¿Cuál es el agente causal del binomio teniasis-cisticercosis en humanos?^1^

¿Cómo se adquiere la teniasis?^2^

¿Cómo se adquiere la cisticercosis humana?^3^

¿Cuál es la forma de infección de los cerdos?^4^

¿Cuáles son las características de la carne de cerdo infectada con cisticercos?^5^

¿La teniasis y la cisticercosis son problemas de salud que afectan a personas de las zonas rurales o urbanas?^6^

Especifique cuál enfermedad grave es causada por teniasis o cisticercosis^7^

Defina brevemente la neurocisticercosis^8^

¿Cuáles son los síntomas y los signos de la cisticercosis?^9^

¿Qué medicamentos genéricos se utilizan para tratar la cisticercosis?^10^

¿Cuáles son los síntomas y los signos de la teniasis?^11^

¿Qué medicamentos genéricos se utilizan para tratar la teniasis?^12^

¿Cuál es el método de elección para el diagnóstico de teniasis?^13^

Parte II: actitudes relacionadas con la teniasis y la cisticercosis

¿Se debe brindar más información al público sobre teniasis y cisticercosis? Sí_No_

Si usted ya conoce cómo se transmite la teniasis y la cisticercosis, ¿ha informado alguna vez a sus allegados cómo se puede transmitir? Sí_No_

Si observa a un familiar, amigo o conocido consumiendo carne de cerdo mal cocida, ¿cuál es su proceder?_____________

Si como bioanalista observa un huevo de *Taenia* spp. en la muestra de un paciente, ¿cuál es su proceder con el paciente infectado?_____________________________

En las comunidades rurales, ¿qué se puede hacer para prevenir la transmisión de la teniasis y la cisticercosis?

En las comunidades urbanas, ¿qué se puede hacer para prevenir la transmisión de la teniasis y la cisticercosis?

Parte III: prácticas asociadas con la teniasis y la cisticercosis

¿Consume carne de cerdo? Sí_No_

¿Cómo la consume? Cruda___Semicruda____Cocida___Bien cocida___

¿Compra usted carne de cerdo cruda o cocida en establecimientos ambulantes? Sí_No_

¿Usted se lava las manos en las siguientes situaciones?

¿Después de ir al baño? Sí_No_

¿Antes de comer? Sí_No_

¿Antes de preparar alimentos? Sí_No_

¿Lava correctamente verduras, frutas y legumbres antes de consumirlas? Sí_No_

¿Qué tipo de agua consume?

Potable___Clorada___Filtrada___Hervida___De chorro___

Como bioanalista, ¿reporta usted a las entidades de salud del estado aquellos casos en los cuales observa huevos de *Taenia* spp.? Sí______________________No

## Anexo 2.

Respuestas posibles a preguntas abiertas de conocimiento sobre teniasis y cisticercosis

^1^ *Taenia solium*

^2^ Consumo de carne de cerdo infectada con cisticercos de *T. solium*

^3^ Ingestión de huevos de *T solium*

^4^ Ingestión de heces contaminadas con huevos de *T. solium*

^5^ Presencia de metacestodos o vesículas hialinas o blanquecinas

^6^ Sí, ambas, principalmente rural

^7^ Sí, neurocisticercosis

^8^ Localización de cisticercos de *T. solium* en el sistema nervioso central

^9^ “Los signos dependen de la ubicación del cisticerco de *T. solium*”-, [se pueden identificar] “por resonancia magnética o tomografías”; “imagen de cisticercos en el sistema nervioso central, hipertensión endocraneana”; “nodulos en piel, cisticercos en el globo ocular”; “síntomas diversos: cefaleas, convulsiones, mareos, etc.”.

^10^ "No hay un tratamiento general”; “tratamientos específicos controlados por neurólogos (antihelmínticos, esteroides, cirugías, entre otros)”.

^11^ “Expulsión de proglótides y huevos”; “síntomas gastrointestinales, escasos”.

^12^ Praziquantel, niclosamida

^13^ Coprología: tamizaje de heces en búsqueda de proglótides y huevos de *Taenia* spp.

Respuestas posibles a preguntas abiertas sobre actitudes positivas respecto a la teniasis y la cisticercosis

^1^ Informar sobre los riesgos para la salud por la infección con cisticercos de *T. solium*.

^2^ Informar al paciente dónde puede conseguir el tratamiento adecuado.

^3^ Evitar defecar al aire libre y la cría de cerdos sin confinamiento; se deben tener medidas de higiene personal básicas.

^4^ Evitar el consumo de cerdo mal cocido y de vegetales sin lavar; se deben tener medias de higiene personal básicas.
